# Identification of prevalence of musculoskeletal disorders and various risk factors in dentists

**DOI:** 10.1016/j.heliyon.2023.e23780

**Published:** 2023-12-23

**Authors:** Vibha Bhatia, Rahul O. Vaishya, Ashish Jain, Vishakha Grover, Suraj Arora, Gotam Das, Anshad M. Abdulla, Shan Sainudeen, Ahmed Babiker Mohamed Ali, Priyanka Saluja

**Affiliations:** a Punjab Engineering College (Deemed to be University), Chandigarh, India; b Dr. H.S.J. Institute of Dental Sciences, Panjab University, Chandigarh, India; c Department of Restorative Dental Sciences, College of Dentistry, King Khalid University, Abha 61421, Saudi Arabia; d Department of Prosthodontics, College of Dentistry, King Khalid University, Abha 61421, Saudi Arabia; e Department of Pediatric Dentistry & Orthodontics, College of Dentistry, King Khalid University, Abha 61421, Saudi Arabia; f Department of Dentistry, University of Alberta, Edmonton, Alberta, Canada

**Keywords:** Musculoskeletal disorders, Dentists, Ergonomics, Nordic questionnaire, Liker scale, Discomfort level, Upper extremity

## Abstract

**Purpose:**

The awkward and repetitive movements lead to tissue straining, potentially leading to painful musculoskeletal disorders (MSDs). MSDs in dentists result in work inefficiency and a reduction in work hours. A survey was conducted to assess the prevalence of MSDs amongst the dental population of interest.

**Methods:**

Customized individual detail questionnaires, Standard Nordic Musculoskeletal questionnaires, and Level of Pain estimation using the Likert Scale were used to deduce the various responsible risk factors for the occurrence of MSDs in dentists. Inferential statistical analysis was done to identify the prevalence and severity of the MSDs. The Chi-Square test (95 % confidence interval) was used to identify and compare the association of risk factors involved in MSDs with the occurrence of the Effect of MSDs, the presence of MSDs, and the severity of the MSDs.

**Results:**

The results of the study deduced that the dentists followed the sedentary work practices. The dentists experienced maximum discomfort in the neck region, which was accompanied by the discomfort experienced in the lower back, hands and wrists, making the upper extremity being more susceptible to the MSDs. Gender risk factors the, the prevalence of MSDs in the dentist's upper back, and the severity of pain in the upper back region showed a significant association level.

**Conclusion:**

The wrist posture, the prevalence of MSDs and the severity of pain in the dentists' neck, shoulder and upper back showed a significant association level.

## 1 Introduction

Awkward postures, repetitive movements, and workplace tiredness often trigger occupational disorders. Different terminologies are used for occupational disorders all over the world. United States (US), Canada and Europe refer to these as Repetitive Strain Injuries (RSI), complications in the neck, arms and shoulders in Brazil, the Netherlands and Australia named it Occupational Overuse Syndrome and Japanese name for it is Cervicobrachial Occupational Disorders [1]. The USA spends approximately two billion dollars annually on its population suffering from Musculoskeletal Disorders, rs (MSDs), usually detected in the neck, arms and shoulders [2]. Sickness leaves at the workplace indirectly reflects the productivity and economy of the country. Assessing the prevalence of the MSDs among workers is necessary to take necessary preventive actions [1].

Workers belonging to both organized and unorganized sectors are affected by the MSDs, but all in different ways. Different body parts are affected differently by different job demands. Goldsmith workers of Karnataka, India, not only suffer from disorders related to the neck (90 %), back (75 %) and wrist (45 %) but also from hazards related to poor illumination such as red-eyed, eye burning, headaches and blurred vision. 24 % and 16 % of the sewing machine workers in the garment industry suffered from neck and distal upper extremity disorders, respectively. The taxi drivers felt pain in the right shoulder (48.3 %), right upper arm (9.2 %) and right forearm (1.7 %) in the last 12 months; this is due to the involvement of upper extremity while doing driving tasks. The mid-backsanitation workers' upper arms, neck, upper back, mid-back and buttocks body area were majorly affected by pains and discomfort [3].

The thumbs and fingers of the meat packers and butchers are highly affected by the continuous involvement of the hands while doing their job. Tractor and subway operators, truck and bus drivers, nurses, warehouse workers, baggage handlers and grocery cashiers are prone to numbness or shooting pain and lower back pain [4].

In some studies, at least 77 % of the dental professionals reported the occurrence of MSDs. Quick and repetitive nature of motion, awkward postures, maximum flexion and extension of fingers and wrists, pressure on tendons, the static compression of muscles, limb nerves (upper and lower), joints, neck and shoulder girdle [[5], [6], [7]]. Studies revealed that 47 % of these disorders emerged after 10 and 19 years of professional experience between 30 and 49 years of age group were most affected, and the musculoskeletal pain was associated to the practicing time. Also, approximately one third of the dentists refer MSDs as the responsible factor for early job abandonment [8,9].

The prevalence of MSDs is reported by dental professionals belonging to different parts of the world. A study by Bret and Gorce et al. stated that >60 %, 35–55 % of the dentists experienced MSDs in the lower back and upper extremity regions of the body [10]. The highest prevalence of MSDs in neck (82 %) and lower back region (64 %) was observed in the dentists belonging to the Malaysia with female dentists being more prone to MSDs [11]. The dentists registered with the Australian Dental Association, Queensland Branch reported that 24.6 %, 22.1 % and 21.8 % of the dental practitioners self-reported MSDs in the neck, lower back and shoulder region [12]. The observations made during the study conducted in Iran revealed that 43.4 %, 35.8 % and 25 % of the dentists were suffering from pain in the neck, back/shoulder and wrist regions of the body [13]. A questionnaire survey study reported that dentists are susceptible to the MSDs of various body parts. Study analysis revealed that the 92 % of the dental practitioners who participated in the study experienced pain in the body regions such as neck (47 %), lower back(35 %), fingers(>29 %), hip(23 %), mid back(20 %) and shoulder(20 %) areas of the body [14]. Prophylaxis and ergonomics were suggested as the possible treatment action for the occurrence of MSDs in dentists [15].

India is tackling occupation-related health issues with difficulty. It is estimated that 40 % of all the health-related costs are spent on occupation-related health issues like MSDs in India. Documentation of the prevalence of Musculoskeletal Disorders is not that well elaborated in the literature available. In 2018, according to the National Health Profile, 2.7 lakh dentists were registered with the Dental Council of India (DCI) for a population of 134 crores. Efforts have been made to study the prevalence of MSDs in the Northern region of India [16]. But limited efforts are made to study the relationship between the prevalence and the risk factors involved in dentistry in India. The prevalence of the occurrence of MSDs among dentists in India is not properly documented [17]. Realizing the presence of improper and minimal data, it becomes imperative to do the research advancement in the current field by studying the prevalence of MSDs amongst dentists and its relationship with the risk factors involved in the Indian diaspora.

The study aims at fulfilling mainly two main objectives. The first is to evaluate the prevalence of the various symptoms related to Musculoskeletal Disorders in dental practitioners working in local Indian scenarios. Further, the identification of the risk factors involved in the dental occupation, which were responsible for the occurrence of musculoskeletal disorders.

This paper summarizes the results of the survey of dentists working in the local dental colleges in Chandigarh to check the prevalence of Musculoskeletal Disorders among dentists. The number of dentists who participated in the current study was 120.

Different studies have established that Standard Nordic Questionnaire can be considered a helpful tool in assessing the prevalence of MSDs [[18], [19], [20], [21], [22]].

The Likert scale has been used in the study go assess the severity of MSDs [23].

The customized questionnaire included general, professional and work-related information, considering all these criteria, it is imperative to do a holistic assessment of the risk factors involved.

Customized individual detail questionnaires, Standard Nordic Musculoskeletal questionnaires, and Level of pain estimation using the Likert Scale were used to deduce the various responsible risk factors for the occurrence of MSDs in dentists.

## 2 Methods

The cross-sectional descriptive form of questionnaire study using novel self-designed subject detail questionnaire and customized Nordic Questionnaire was performed. The study by Kuorinka et al., 1987 discussed the customized Nordic Questionnaire in detail [24]. The self-prepared questionnaire was pre-tested and validated against the sample population. As in the study by Saini et al., 2023 [25], assessment and evaluation of the questions was done for internal reliability of the questionnaire using the Cronbach's alpha measure to zanalyze questionnaire's internal consistency. The cronbach's alpha value was acceptable for the sample and was mostly greater than 0.70 in all cases. The validated questionnaire was administered to the subjects who participated in the current study. The survey conducted in this chapter is divided into two sections. The first section of the study evaluated the prevalence of the various symptoms related to Musculoskeletal Disorders in dental practitioners working in local Indian scenarios. The second section of the study included the identification of the risk factors involved in the dental occupation, which were responsible for the occurrence of musculoskeletal disorders. The study results will help in finding out the risk factors and the associated severity which might be responsible for the occurrence of MSDs in dentists. The prior knowledge of MSDs and associated severity becomes imperative to apply any assessment technique in dentistry. Efforts will be made to reduce the occurrence of MSDs in dentists on knowing the prevalence and responsible risk factors of MSDs.

### 2.1 Summary of subjects

This cross-sectional study was conducted in the local dental institute in Chandigarh, India. The 120 dental practitioners belonging to different specialized areas of dentistry participated in the questionnaire study. All the dental practitioners who participated in the study were not suffering from any pre-acquired critical disorder, which could create a hindrance in performing dental practice. The subject's summary has been tabulated in Table 1. Before the study, permission was taken from the institute's head of department and research degree committee. Each participant signed the duly filled informed consent form. The complete data collection procedure was explained to the participants, and any doubt to them in the questionnaire was seriously clarified. Primary anthropometric measures such as the weight and height of the participants were tabulated using a digital weight measurer and manual stadiometer.

**Table 1:** Summary of physical parameters of the dental practitioners participating the study.

Sr. No.	Parameters	Range	Mean (Standard Deviation)
1.	Weight(kg)	40–88	64.03(8.47)
2.	Height(cm)	150–178	165.13(5.67)
3.	Age(years)	22–67	33.75(24.93)
4.	BMI	12.62–39.11	25.86(13.25)

### 2.2 Experimental protocol

The investigation using questionnaires included the data variables related to general, related, and professional information like basic anthropometric measures, lifestyle habits, professional qualifications and specialization, work experience details, breaks during work, exercise schedule, MSDs awareness, and other related information.

#### 2.2.1 Section I

The self-designed subject detail questionnaires included three kinds of Information details and the associated questions, both of which are described as follows.

1. General Information
• Physiological details like Age, Gender, Weight, Height, Dominant Hand
• The marital status of the subject
• The habits like Smoking, Alcoholism, and Physical Recreational Activity of the subject
2. Profession Related Information
• The highest qualification of the subject (e.g., BDS/MDS/Dental Hygienist Course)
• The subject is working at what position professionally, working as UG student or PG student or Intern or Dental Hygienist or Professor or Dentist
• The specialization of the subject if they have already completed masters, the subject is specialized in Prosthodontics or Periodontics or Endodontics or Orthodontics or Pedodontics or Oral Medicine & Radiology or Community Dentistry
• The number of years of dental practice undergone by subject till date (in years; including the time period of the BDS internship)
3. Work Environment Information
• Working shift related information, doing a single shift or more than one shift, duration of the working shift, rotation of Job permissible while working
• The break time is taken after completing one procedure, 5–10 min after every patient or 5–10 min break is taken after every few hours or do not take a break if patients are waiting
• Type of activity if performed by the dentist during the break duration, like stretching exercise or sitting or lying of dental chair or walking in the corridor
• Working position of the dentist, like standing position or sitting position or standing most of the time or sitting most of the time
• The frequently adopted posture by the dentist while working, like if the pose is straight or bent body posture is adopted, or the posture of the dentist is both bent and twisted
• The most common status of the wrist of the dentist while working, like flexed at 90 or flexed and rotated or relaxed
• The existence or non-existence of mental stress while performing dental work
• The frequency of exercises performed by the dentist, twice daily or once daily or 3–4 times a week or occasionally or rarely or never
• The types of exercises performed by the dentist in routine, like walking or jogging or running or swimming or gym or yoga or any other
• The dentist has attended any educational or awareness program regarding MSDs or not
• The dentist has benefitted from the educational program regarding MSDs or not
• The effect of MSDs on different aspects of the life of dentists, like personal aspects or social aspects or the need to do or compromise with the dental practice.

The Standard Nordic Questionnaire was used to deduce whether the subjects experienced any symptoms related to musculoskeletal disorders, such as pain, numbness, ache, and discomfort in any body part during the last 12 months.

Further, a Likert scale rating was given by subjects to estimate the level of pain in various body parts, which reported musculoskeletal disorders symptoms in subjects.

#### 2.2.2 Section II

The second section of the study included identifying the risk factors involved in the dental occupation that were responsible for the occurrence of musculoskeletal disorders.

The data collected in Nordic questionnaires and Likert Scale scores was observed, re-organized, statistically analyzed, and results were recorded. To identify the prevalence of severity of the MSDs, inferential statistical analysis was done using the SPSS software. To identify and compare the association of the risk factors involved in musculoskeletal pain with the occurrence of Effect of MSDs, the presence of MSDs, and the severity of the MSDs (from the Likert Scale scores) related to various body parts of the dental practitioners which were experienced in last 12 months, a Chi-Square test with a confidence interval of 95 % was used.

## 3 Results

### 3.1 Results of section I

The results of section I related to the self-designed subject detail questionnaires having general and work-associated information are summarised in Table 2.

**Table 2:** Summary of self-designed subject detail questionnaire results.

General Information results	
Dominant hand of usage	•Right Hand (95.83 %) •Left Hand (4.166 %)
Marital status	•Married (51.66 %) •Single (47.5 %)
Lifestyle habits	•Occasional smoking (9.17 %) •Regular smoking (1.67 %) •Never Smoked (90 %) •Occasional Alcoholism (14.17 %) •Regular Alcoholism (21.67 %) •Never had Alcohol (64.17 %)
Physical Recreational Activity	•Gym (10 %) •Running (10.83 %) •Walking (6.67 %) •Swimming (0.83 %) •Yoga (2.5 %) •No Activity (69.17 %)
Profession related information	
Highest Qualification	•BDS (60.83 %) •MDS (30.83 %) •Dental Hygienist course (8.33 %)
Job Title	•Professors (12.5 %) •Dentists (29.17 %) •Dental Hygienists (8.33 %) •Interns (22.50 %) •UG students (12.50 %) •PG students (13.33 %) •Research Associates (1.67 %)
Specialization	•General dentistry (62.50 %) •Periodontics (21.67 %) •Orthodontics (6.67 %) •Endodontics (8.83 %) •Oral Pathology (0.83 %)
Years of Practice	•<1 years (15 %) •1–5 years (35 %) •6–10 years (9.17 %) •11–15 years (10 %) •16–20 years (21.67 %) •21–25 years (7.50 %) •26–30 years (1.67 %)
Shift Duration	•<3 h (60 %) •to 6 h (39.17 %) •7–9 h (0.83 %)
Break within shifts	•5–10 min (75 %) •No break (except lunch break) (25 %)
Activity performed in breaks	•No activity between the breaks (70 %) •Walking in corridor (21.67 %) •Stretching (8.33 %)
Working Position	•Sitting (75.83 %) •Mostly sitting (17.50 %) •Standing (5 %) •Mostly standing (1.67 %)
Common working posture	•Bent back (55.83 %) •Straight back (27.50 %) Bent and twisted back (16.67 %)
Mental work pressure	•Yes (29 %) •No (71 %)
Frequency of Work-out	•One or other type of workout once daily (33.33 %) •3–4 times/week (32.50 %) •Rarely and occasionally (9.17 %)
Effect of MSDs	•Personally affected (29.17 %) •Had to a compromise with the dental practice in one or another form (21.67 %) •No substantial effect on their lives(49.17 %)

Standard Nordic Musculoskeletal questionnaire and Likert scale rating results.

At which body parts did the subjects experience pain or discomfort?

**Fig. 1:**
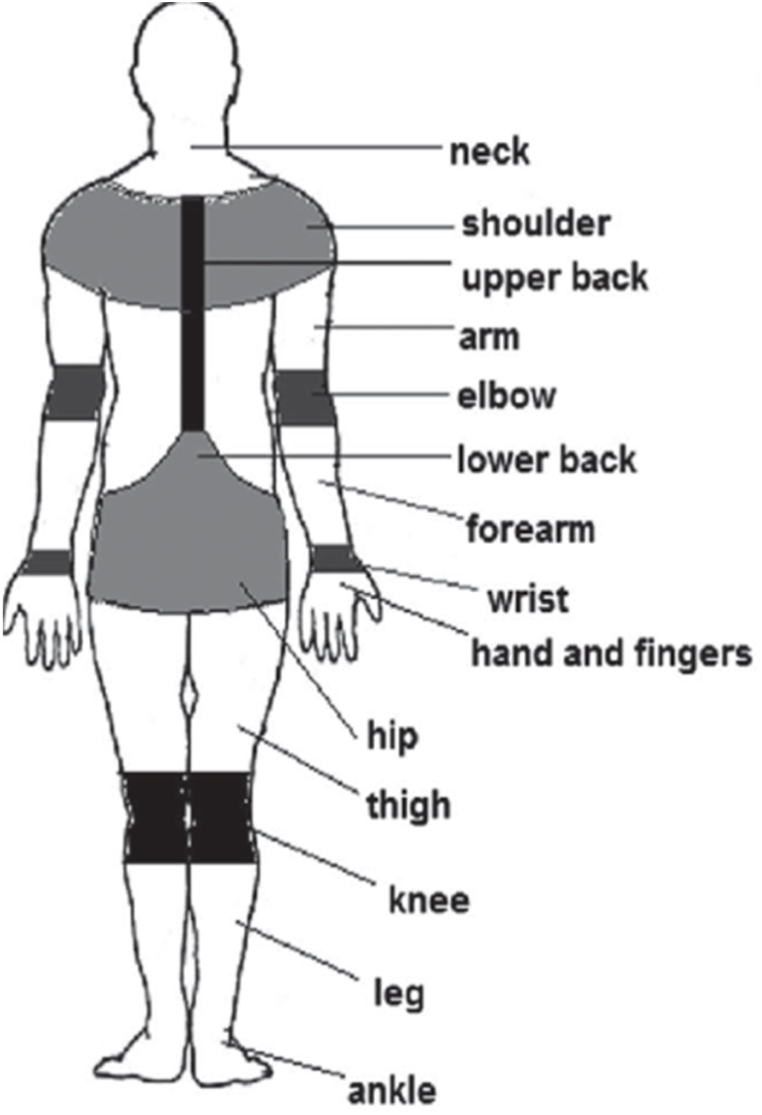
Exact location of the body parts experiencing pain or discomfort.

**Fig. 2:**
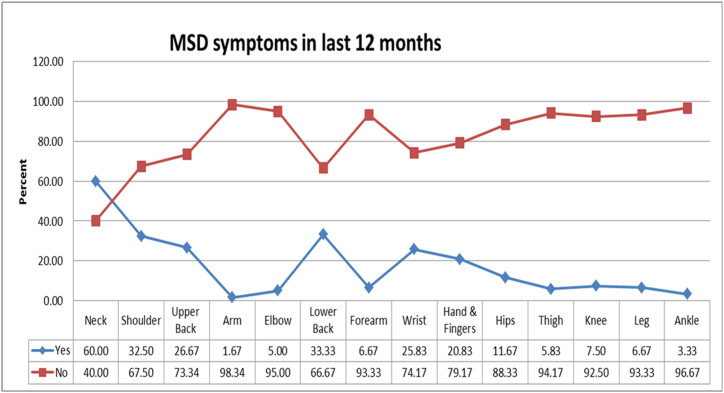
Percentage of MSDs symptoms observed in various body regions during the last 12 months How bad is the pain.?.

The perceived pain data for the past few months was collected quantitatively by using Standard Nordic Musculoskeletal questionnaires for body parts like neck, shoulder, upper back, arm, elbow, lower back, forearm, wrists, hand & fingers, lower back, hip, thigh, knee, leg, and ankle. The exact location of the body parts is represented in Fig. 1. Out of the 120 dentists (subjects) who admitted that they felt pain, a maximum number of subjects experienced pain/discomfort in the neck region (60 %) in the past 12 months. Further, 33 % of the subjects felt pain/discomfort in their lower back, followed by 32.50 %, 26.67 %, 25.83 %, 20.83 %, 11.67 %, 7.50 %, 6.67 %, 6.67 %, 5 %, 3.33 %, and 1.67 % of subjects experiencing pain in shoulder, upper back, wrist, hand & fingers, hips, knees, forearms, legs, elbow, ankle and arms. Graph shown in Fig. 2 shows the existence of pain/discomfort in the subjects.

**Fig. 3:**
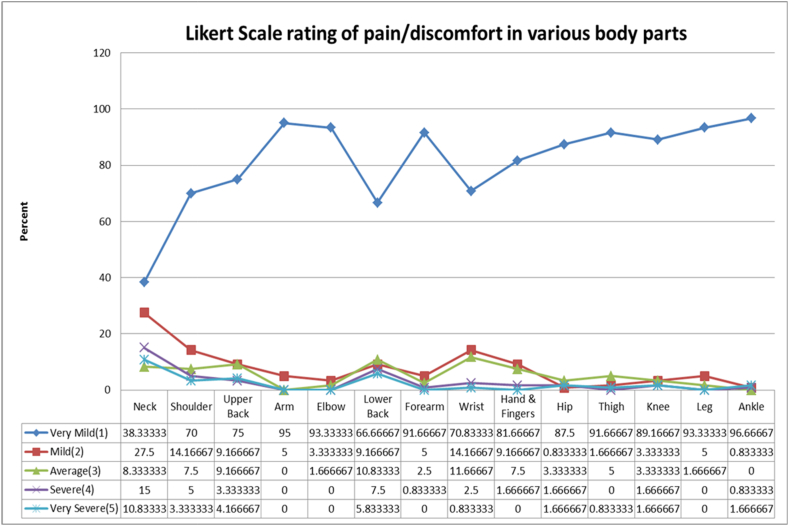
Percentage of Likert scale rating of pain/discomfort in various body regions.

The graph shown in Fig. 3 represents the results obtained from the Likert scale rating. It was observed that 10.83 % and 5.83 % of the subjects experienced severe pain in the neck and lower back, followed by 4.16 %, 3.33 %,1.67 %,1.67 %,1.67 %,0.83 %, and 0.83 % subjects experiencing severe pain in the upper back, shoulder, hip, knee, ankle, wrist, and thigh respectively. Also, 15 % of the subjects suffered severe pain in the neck, followed by 7.5 % of subjects feeling pain in the lower back. Ankle, arm, elbow, leg, forearm, thigh, knee, and hip with 96.66 %, 95 %,93.33 %, 93.33 %, 91.66 %, 91.66 % of the subjects experiencing very mild pain irrespective of the prevalence of their occurrence.

### 3.2 Results of section II

The results from the Chi-Square test performed to check the association of the risk factors involved in musculoskeletal pain with the occurrence of MSDs and the presence of MSDs is summarised in Table 3. Also, the results from the Chi-Square test conducted to check the association of the risk factors involved in MSDs with the severity of MSDs related to various body regions of dentists is summarised in Table 4.

**Table 3:** Results from the Chi-Square test conducted to check the association of the risk factors involved in MSDs with the severity of MSDs related to various body regions of dentists.

Ankle	0.64	0.15	0.84	0.33	0.57	0.07	0.29	1.00	0.02	1.00	0.60	0.07	0.02
Leg	0.12	1.00	0.44	0.37	0.02	0.00	0.10	0.43	1.00	0.11	0.91	1.00	0.22
Knee	0.60	1.00	0.33	0.55	1.00	0.39	0.65	0.45	0.11	0.23	0.52	0.45	0.26
Thigh	0.01	0.70	0.82	0.20	0.37	0.01	0.00	0.67	0.07	1.00	0.27	1.00	0.03
Hip	0.00	0.48	0.95	0.03	0.01	0.00	0.00	0.23	0.01	0.31	0.06	0.48	0.72
Hand&Fingers	0.54	0.50	0.71	0.00	0.00	0.01	0.02	0.23	0.01	0.31	0.04	0.48	0.72
Wrist	0.05	1.00	0.89	0.02	0.05	0.05	0.00	0.37	0.20	0.35	0.05	1.00	0.67
Forearm	0.12	0.47	0.49	0.93	1.00	0.24	0.27	0.43	0.09	0.20	0.11	0.69	0.21
Lower Back	0.24	0.33	0.92	0.09	0.18	0.05	0.16	1.00	0.11	0.12	0.47	0.06	0.19
Elbow	0.03	0.23	0.84	0.03	0.64	0.02	0.15	1.00	0.35	0.04	0.01	0.67	0.04
Arm	0.83	1.00	0.25	0.36	1.00	0.72	0.59	0.51	1.00	0.45	0.08	0.50	0.27
Upper Back	0.26	0.05	0.82	0.14	0.64	0.34	0.30	0.37	0.21	0.10	0.09	0.01	0.70
Shoulder	0.14	0.01	0.11	0.02	0.37	0.02	0.25	0.09	0.01	0.04	0.73	0.05	0.08
Neck	0.42	0.09	0.18	0.08	0.52	0.07	0.03	0.42	0.14	1.00	0.77	0.03	0.31
Effect of MSDs	0.14	0.82	0.49	0.26	0.05	0.08	0.04	0.09	0.94	0.30	0.11	0.70	0.90
Risk Factors	Age	Gender	Specialization	Work hrs	Break	Job Title	Years of Practice	Break Activity	Working Position	Common Posture	Wrist Posture	Mental Stress	Exercise Frequency

Also, the results from the Chi-Square test conducted to check the association of the risk factors involved in MSDs with the severity of MSDs related to various body regions of dentists are summarised in Table 4.

**Table 4:** Results from the Chi-Square test conducted to check the association of the risk factors involved in MSDs with the severity of MSDs related to various body regions of dentists.

DL Ankle	0.93	0.24	0.95	0.45	0.50	0.43	0.84	0.62	0.37	0.21	0.47	0.04	0.35
DL Leg	0.24	0.32	0.64	0.71	0.06	0.02	0.42	0.48	0.56	0.46	0.65	0.64	0.50
DL Knee	0.71	0.92	0.74	0.80	0.49	0.76	0.52	0.17	0.14	0.59	0.36	0.12	0.15
DL Thigh	0.05	0.04	0.98	0.87	0.31	0.35	0.11	0.44	0.08	0.25	0.35	0.77	0.12
DLHip	0.00	0.31	0.72	0.57	0.17	0.64	0.02	0.39	0.24	0.06	0.10	0.66	0.22
DL Hand &Fingers	0.09	0.98	0.83	0.00	0.00	0.00	0.03	0.07	0.03	0.19	0.02	0.40	0.80
DL Wrist	0.00	0.21	0.66	0.03	0.02	0.00	0.00	0.45	0.27	0.92	0.03	0.70	0.18
DL Forearm	0.68	0.22	0.96	0.83	0.11	0.75	0.53	0.40	0.41	0.15	0.18	0.59	0.58
DL Lower Back	0.01	0.38	0.96	0.01	0.06	0.02	0.01	0.86	0.00	0.57	0.83	0.06	0.28
DL Elbow	0.05	0.09	0.82	0.05	0.03	0.29	0.12	0.61	0.59	0.06	0.09	0.32	0.19
DLArm	0.50	0.45	0.40	0.04	0.17	0.57	0.64	0.67	1.00	0.18	0.50	0.06	0.18
DLUpper Back	0.07	0.02	0.14	0.53	0.09	0.25	0.06	0.57	0.15	0.50	0.08	0.02	0.94
DL Shoulder	0.06	0.23	0.46	0.46	0.18	0.22	0.01	0.41	0.36	0.11	0.11	0.01	0.73
DL Neck	0.00	0.31	0.06	0.03	0.00	0.00	0.00	0.75	0.05	0.14	0.28	0.03	0.33
Risk Factors	Age	Gender	Specialization	Work hrs	Break	Job Title	Years of Practice	Break Activity	Working Position	Common Posture	Wrist Posture	Mental Stress	Exercise Frequency

The statistical analysis showed some association of significance (p < 0.05) between the risk factors of musculoskeletal disorders and some of the highest musculoskeletal disorders prevalence in the last 12 months among the body parts such as the neck, lower back, shoulder, upper back, wrist and hand & fingers (shown in Table 3). The overall effect of MSDs observed on the dentists showed a significant association with the break between the shifts and the years of dental practice. The neck pain has 60 % prevalence (highest of all) was significantly associated with the Years of Practice (p = 0.03). Prevalence of neck, shoulder, and upper back disorders was associated with the mental stress experienced by dentists. Gender was seen associated with the upper back and shoulder-related MSDs. It was also observed that the shoulder, wrist, and hand & fingers showed association with the continuous number of work hours performed by the dentists. In the current study, the dentist's wrist posture had a significant association with elbow, wrist, hand & fingers. Work breaks showed significant association with the wrist and hand & fingers. Exercise frequency did not show any significant association except with the elbow, thigh, and ankle. Any kind of physical activity performed between the shifts did not show any significant association with the prevalence of musculoskeletal pain in any body part of dentists. Only the shoulder and elbow were significantly associated with the common posture adopted by the dentists. Years of the practice of dental practitioners were significantly associated with the neck, wrist, hand & fingers among the highly prevalent MSDs. It was noted that the type or title of the job had significantly associated results with the body parts like shoulder, lower back, wrist, hand & fingers among the highly prevalent MSDs and also with the elbow, hip, and thigh regions of the body. However, the area of specialization of the dental field did not have any significant association with any prevalence of MSDs in any of the body regions.

The statistical analysis showed some association of significance(p < 0.05) between the risk factors related to MSDs in dentists and the severity of MSDs among the body regions showing the highest prevalence of MSDs like neck, lower back, shoulder, upper back, wrist, and hand & fingers (shown in Table 4). It was observed that for the maximum number of the MSD risk factors, the severity of MSDs in the neck, wrist, and hands & fingers body regions showed an association. For age factors, the severity of MSD pain in the neck, lower back, and wrist (amongst the areas of high prevalence) showed the association. The specialization area of dentists was not associated with the severity of MSDs in any of the dentist body parts. Continuous working hours also showed association with the neck, lower back, wrist, hand & fingers. The break taken within the shifts risk factor was associated with the severity of MSDs in the neck, wrist, elbow, and hand & fingers. The job title or designation of the dental practitioner showed association with the severity of MSD pains in the neck, lower back, wrist, hand & fingers, and legs. The experience of dental practice in years did show some association with the MSD severity in the neck, shoulder, lower back, wrist, hand & fingers, and hip body regions. Any activity performed during the break time, the common dental postures, and the exercise frequency showed no single association with the MSD severity of any of the body regions of the dentists. The position in which the dentist performed sitting or standing had an association with the severity of MSDs in the body regions such as the neck, lower back, and hand & fingers. The association test results of the wrist posture risk factor showed association with the wrist and hand & fingers. The mental stress risk factor was also seen associated with the severity of MSDs in body parts like the neck, shoulder, and upper back (from the body parts having the maximum prevalence of MSDs).

## 4 Discussion

### 4.1 Section I

One hundred and twenty subjects participated in the questionnaire study. The questionnaire study was divided into two sections. The first section included the general information analysis and the prevalence of musculoskeletal disorders in the subjects (dentists) who participated in the study, which was fulfilled using self-designed subject detail questionnaires, standard Nordic questionnaires, and body pain assessment using a Likert scale rating.

Further, the first part of the self-designed subject detail questionnaires gathered general information about the subjects who participated in the study. A large number of participants (95.83 %) were using the right hand as the dominant hand. The results suggested that an almost equal number of participants were holding married and single status, which is 51.66 % and 47.5 %, respectively. The study may not prove as effective to find married/single parameters to affect the prevalence of MSDs in subjects. Alcoholism was relatively more prevalent among participating dentists with higher percentages of occasional (14.17 %)and regular(21.67 %) drinkers if compared with other lifestyle habits such as smoking(9.17 % of regular smokers). The number of alcoholic undergraduate dental practitioners in UK was quite high and was approximately estimated it to be 85 %, quite less than the number reported in the current study. The UK study also reported 23.6 % male and 12.2 % female dentists having tobacco smoking habits, again higher in number than reported in the current study [26]. With 10 %, 10.83 %, 6.67 %, 0.83 %, and 2.5 % of the subjects performing gym exercises, running, walking, swimming, and yoga, respectively in routine, shows most of the dentists still followed a sedentary lifestyle. The outcomes of our study are similar to the study conducted by Singh and Purohit, which revealed that most of the dentists suffered from obesity and practiced minimal physical activity [27]. The reduction in physical activity is usually because of the long working hours in dental clinics, operated mainly by dentists as the private practice in the evening after they work in dental college during the day.

The second part of the self-designed questionnaires gathered professional information about the subjects who participated in the study. A maximum number of dentists who participated in the study had completed bachelor's (60.83 %), and nearly one-third of them had done specialization in some field of dentistry (30.83 %). There was a special branch portion of the participants who had neither done bachelor's and master's but were working as dental hygienists after completing the specialized course in dental hygiene(8.33 %). The data was supported by the survey study by a Government Dental College in which 70 % of the final year dental students in India were interested in pursuing post-graduation [28]. Nearly one-third of the subjects amongst the subjects were practicing general dentistry (29.17 %); the rest others included professors, interns, dental hygienists, UG and PG students, and researchers. The participants involved in a variety of job roles assisted in finding out if the prevalence showed any dependency upon the type of job role. Further, apart from 62.50 % of participants practicing general dentistry, dentists belonging to different specializations participated in the study. Most of the participants in the study were relatively young age, and the least percentage of the participants (1.67 %) had 26–30 years of work experience category and belonged to the older age group category. Nearly 40 % of the participants followed nearly 4–6 h of continuous sitting while working. Such long duration of working hours in dental occupation results in muscle strain and other musculoskeletal disorders in dentists. In the current study, only 25 % of the participants reported that they worked continuously without breaks between the continuous sitting shifts. In the current study, most of the participants who took a break between the shifts (70 %) did not perform any physical activity during the break time. Dentists might have felt like taking a rest by sitting at one place for break time as well. The rest of them did walking and stretching activities between the breaks. Sitting was the most prominent working position reported among participants (nearly 93 %, including dentists who followed mostly sitting but rarely standing work position norm). 55.83 % of the dentists adopted the bent back posture, and it was the most commonly adopted posture among all the dentists compared with the straight back working posture adopted by 27.50 % of the dentists. Bent back with a twist angle was reported in 16.67 % of the dentists. This fact is supported by the study in which 26.8 % of the dentists maintained bent back to the right side for more than the 60s [29]. In the dental profession, doctors experience mental pressure, as 71 % of the dentists reported a stressful professional environment. In a study, it was mentioned that nearly half of the dental practitioners felt that stress factor during work situation affects their work efficiency. The study published in the British Dental Journal claims that the mental trauma in dentists is mostly due to fear of legal regulations and complaints from patients [30]. It is a noticeable point that despite the fact that a large portion of the dentists who had attended a minimum of one MSD related seminar till now are still suffering from musculoskeletal disorders.

Among all the body parts, the neck region seemed to experience discomfort most of the time and even received the highest score on the Likert scale, making it susceptible to the highest discomfort level. The other studies also supported the occurrence of a higher prevalence of neck pain in dentists [31]. The neck muscles are overused, and active muscles become prone to injury, resulting in inflammation. The prevalence of pain might be high in the neck because dentists need to make awkward movements of the neck to have a proper look inside the oral cavity of the patients while working. After neck and lower back muscles were prone to muscle disorders in dentists due to working conditions. About 33 % of the dentists felt some discomfort in their lower back region, with 5.83 % having severe pain. In another study, about 30 % of the dentists felt the same amount of discomfort [32]. The prolonged spinal bend and extension of the torso in the patient's direction and maintaining the posture for longer duration causes over-exertion of the lumbar and cervical region of the spine. Limited working space and to have a look into the oral cavity repeatedly performing tender procedures using tiny instruments causes overburdening of the muscles, ligaments, small joints, and tendons [33]. From the data, it is observed that major contribution of discomfort in dentists is experienced in body parts like the neck, lower back, upper back, shoulders, hands and wrist regions. As 20.83 % of the dentists are experiencing hand and wrist-related problems, this elevates the chances of occurrence of cumulative trauma disorders like Carpal Tunnel Syndrome. As the dental profession includes repeated stress on the hand and wrist region due to movements, it results in inflamed tendons and compressed median nerve. Mostly canal cleaning and exfoliation jobs are responsible for such problems. It was generally observed from the data that the body parts except upper extremity are comparatively less susceptible to MSDs. Body parts like hips, knees, legs and ankle are not only less prevalent (with less than 15 %) among the subjects but also the severe symptoms were least in number (less than 2 %). This may be due to the low number of frequent movements involved with the lower extremity body parts during dental practice. The susceptibility of the upper extremity to repetitive and continuous movements for a longer duration may be the reason for more severity of observed symptoms.

### 4.2 Section II

The Nordic questionnaire study on one hundred and twenty dental practitioners was followed by the MSDs and their severity rating on the Likert scale. Some of the results of the study summarised in Table 3, Table 4 coincided with similar previous studies conducted. The gender risk factor was seen to be significantly associated with the prevalence of MSDs in the upper back and shoulder regions of the dentist's body and further with the discomfort level in the upper back region from the Likert scale study. Current and earlier studies have reported a higher prevalence of MSDs among females [34]. This is probably due to gender-related physical and physiological differences. It is also evident that the female muscles can exert just two-third of the male muscle force, making body stabilization difficult. The study observed a higher prevalence of pain in the lower back, shoulder, hands, hips, and neck region of the female dentists [35]. The results of another study coincided with the results of the present study; it reported a significantly higher prevalence of pain in the shoulders in female dentists [36]. In the present study, the age factor was not significant in the prevalence of MSDs among the earlier defined high MSDs prevalent body regions in dentists. It showed significant prevalence as well severity level in the other body regions such as elbow, wrist, hip, and thigh. The continuous working hours for the dentists showed a significant association with the prevalence of pain in the body regions like shoulder, wrist, hand & fingers, and other body regions. Hand & fingers reported a significant association with the severity of the pain. It is seen that the damaged tissue gets repaired while resting. If the damage rate overtakes the rate of resting intervals, it imparts the physical load on the body and leads to MSDs. Dental practitioners give importance to the idea of the continuous work habits leading to musculoskeletal pain in prominent body regions [[27], [28], [29], [30], [31], [32], [33], [34], [35], [36], [37], [38]]. The job title risk factor was associated with the shoulder, lower back, wrist, hand & fingers among the prevalent body regions of the dentists. A study revealed that due to the static nature of the work with forceful and repetitive pinching, periodontists are more prone to experience MSDs in the neck, shoulder, and wrist area [39]. Due to static posture but lower repetitive movements, while working, the general dental practitioners are more prone to discomfort in the neck and lower back. Orthodontists have to lean forward repetitively, and the lower back is often bent while doing clinical activities. In the current study, the exercise regime followed by dentists did not largely to have association with the occurrence of MSDs. This result is in contrast with the other studies where the dentists who performed regular exercises were less likely to experience any MSD [40]. This may be due to the reason that due irregular working hours, some of the dentists may not be regularly following that routine that they have mentioned in the questionnaire. The results of the current study did not show much meaningful association between the working position (standing or seated) with the prevalence and severity in the body parts. This is explainable as the appropriate seating is crucial to perform the precise dental job and gives comfortable working. The seating, if unsupported, not only raises the lumbar pressure but also causes an imbalance between the abdomen and lower back due to an increase in forces in lumbar joints [41]. Therefore, a seated position is not always a solution to eliminating injury or discomfort [42]. The straight or twisted, bent dental posture factor had a significant impact only on the shoulder and elbow region of the dentist. There is a possibility that the dentists, while filling the questionnaire data, did not provide the data as accurately considering the chance of mistakes in the estimation of more time being in a straight or bent posture. A significant association of wrist posture was seen with the prevalence of MSDs in the elbow, wrist, and hand & fingers. The association of the wrist posture was associated with the severity of symptoms only in the elbow region of the dentist's body. The continuous flexion and the extension of the wrist and fingers are usually responsible for MSDs and disorders like CTS, and the resultant pain can make the dentist unable to perform properly [43]. The ergonomic design of dental tools by optimization of design parameters like sharpness, shape, texture, balance, weight, and diameter can help in reducing the muscle load of hands and wrist [42]. The mental stress factor showed a significant association with the prevalence and severity of pain in the neck, shoulder, and upper back of the dentists. Cognitive stress is often associated with the upper extremity and cervical disorders in the related body parts [44]. The stress may be due to extrinsic factors like job demands, workload, job control, financial issues, and time targets [42,44].

### 4.3 Relevance of results and some clinical implications of the study

• Study suggests that the working conditions of dentists mostly affecting neck region followed by lower back muscles of the dentists. This was also observed in the study by Khan et al. [11]. Dental working conditions are fairly affecting body regions such as upper back, shoulders, hands and wrist. The study implies that lower extremity of dentists is least affected by the working conditions and the same was reported in the study [10]. Upper extremity postural correction techniques are expected to assist dental ergonomics.
• It is implied from the current study that due to lower physiological strength in the upper back and shoulder regions of females, they are more prone to MSDs and, therefore, need extra attention for better work and personal life. The higher prevalence of the MSDs in females is supported by the results of other studies [34].
• The continuous working hours and intricate hand and wrist movements in dentists are contributing towards the prevalence of pain in the body regions like the shoulder, wrist, hand and fingers, and other body regions. Other studies have also reported the presence of pains in shoulders, hands and wrist (apart from neck and lower back regions) in dentists due to dental work practice followed by the dentists [45].
• Due to static posture but lower repetitive movements while working, the general dental practitioners are more prone to discomfort in the neck and lower back.
• Study suggests that dentistry involves considerable mental work stress and is associated with severe pains in upper extremity of body parts such as the neck, shoulder and upper back. The stiffness in these body regions are often associated with a cervical problem and needs attention.

### 4.4 Limitations

There are a few limitations to the current study. The questions in the questionnaire study lacked the flexibility for the subjects if in case they wanted to leave some specific comments. The option for making question related particular comments would have formed the strong basis for the questionnaire analysis. Since the questionnaires were distributed in the same sector of subjects, there is the possibility of overlapping of the results due to peer interaction.

## 5 Conclusion

In this study, almost half of the dental population (40 %) practised continuous sitting hours while performing their work (4–6 h), out of which 25 % of the dentists worked without taking any breaks between the shifts. Nearly three-fourths of the dentists who took breaks between the shifts were reluctant to do any physical activity during the break and preferred taking rest instead. Sitting was the most prominent (93 %) working posture followed by the dental practitioners primarily because it provides support to the leg and back of the dental practitioner. This indicates the sedentary behaviour of the dental practice performed by the Indian Dentists. Among all the body parts, primarily the neck region received the highest score on the Likert scale, making it susceptible to the highest discomfort level. The prevalence of neck pain in dentists is followed by the majority of lower back pain in dentists. The 20.83 % prevalence of hand and wrist-related problems promoted the chance of CTDs like CTS. The data revealed the comparatively more susceptibility of the upper extremity body part to MSDs. A significant association level was seen in the gender risk factor and the prevalence of MSDs in upper back and shoulder regions of the dentist's body and further with the discomfort level in the upper back region from the Likert scale study. Significant association level was seen in the wrist posture and the prevalence of MSDs and the severity of pain in the neck, shoulder, and upper back of the dentists.

## 6 Recommendations

In future studies, efforts may be made to overcome the above mentioned limitations for better research results. Comments section for the subjects to leave feedback and suggestion should be added to the questionnaire. If possible, it is suggested to distribute questionnaire to subjects in isolation to eliminate the peer influence factor while filling up the form.

## Declarations

### Conflict of interest

The authors have no relevant financial or non-financial interests to disclose.

The authors have no competing interests to declare that are relevant to the content of this article Institutional Review Board**.**

**Statement** The study was conducted in accordance with the Declaration of Helsinki and approved by the Ethics Committee of Punjab Engineering College (Deemed to be University), Chandigarh, India (PEC/ERC/12/2022/28) and was conducted in agreement with the ethical standards of the declaration of Helsinki.

## Data availability

All data for evaluating the conclusions are presented in this paper. Additional data related to this study can be obtained from the corresponding authors upon reasonable request.

## Funding

The authors extend their appreciation to the Deanship of Scientific Research at King Khalid University, Abha, Saudi Arabia for supporting this work through a Large Research Group Project under grant number (RGP2/198/44).

## Institutional Review Board statement

The study was conducted in accordance with the Declaration of Helsinki and approved by the Ethics Committee of Punjab Engineering College (Deemed to be University), Chandigarh, India (PEC/ERC/12/2022/28) and was conducted in agreement with the ethical standards of the declaration of Helsinki.

## Informed consent statement

Informed consent was obtained from all subjects involved in the study.

## CRediT authorship contribution statement

**Vibha Bhatia:** Writing – original draft, Conceptualization. **Rahul O. Vaishya:** Writing – review & editing, Project administration, Data curation. **Ashish Jain:** Investigation, Formal analysis. **Vishakha Grover:** Resources, Methodology. **Suraj Arora:** Software, Funding acquisition. **Gotam das:** Visualization, Supervision. **Anshad M. Abdulla:** Validation, Resources. **Shan Sainudeen:** Project administration, Data curation. **Ahmed Babiker Mohamed Ali:** Writing – review & editing, Investigation. **Priyanka Saluja:** Project administration.

## Declaration of competing interest

The authors declare that they have no known competing financial interests or personal relationships that could have appeared to influence the work reported in this paper.

## Figures and Tables

**Figure:**
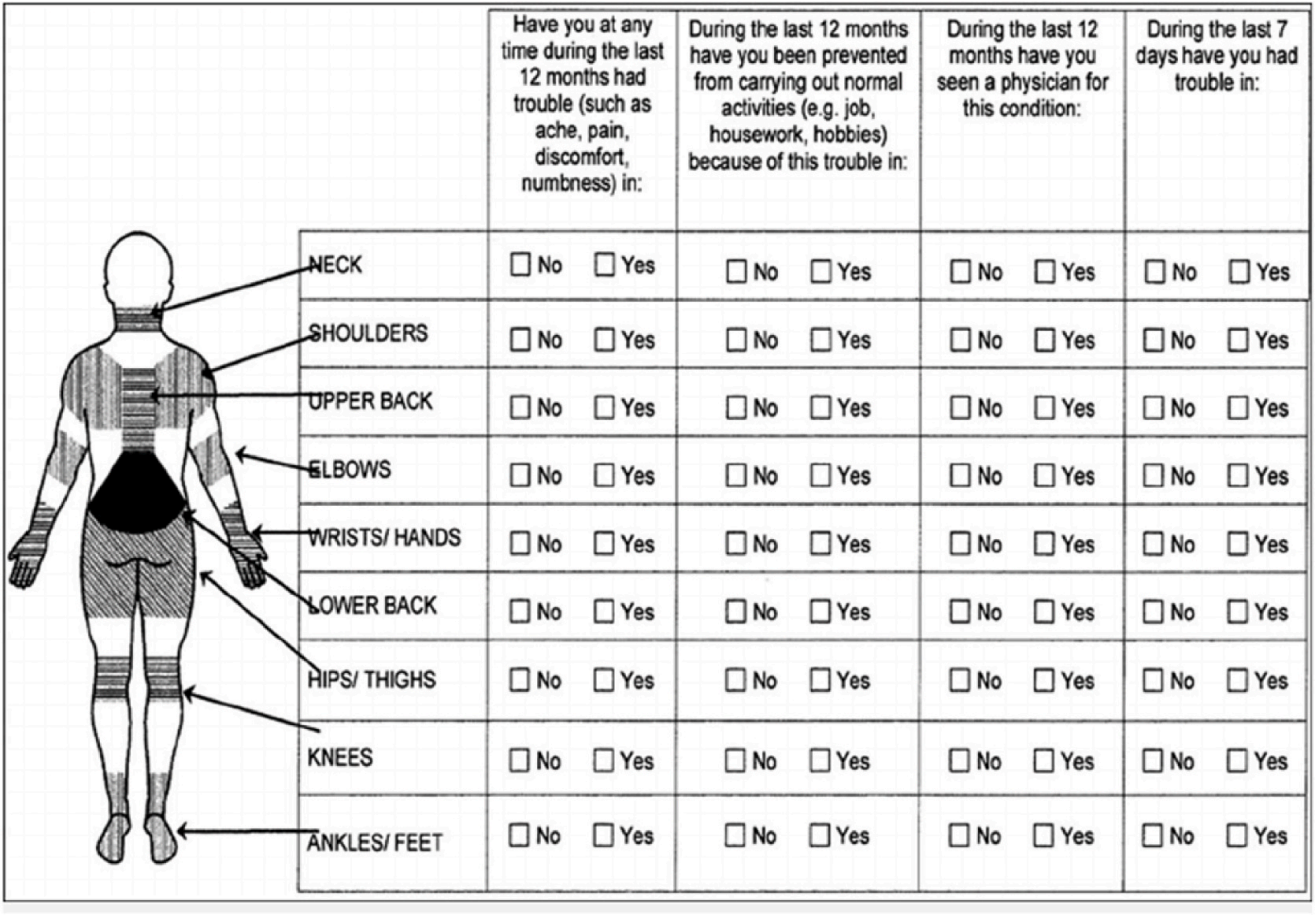


**Figure:**
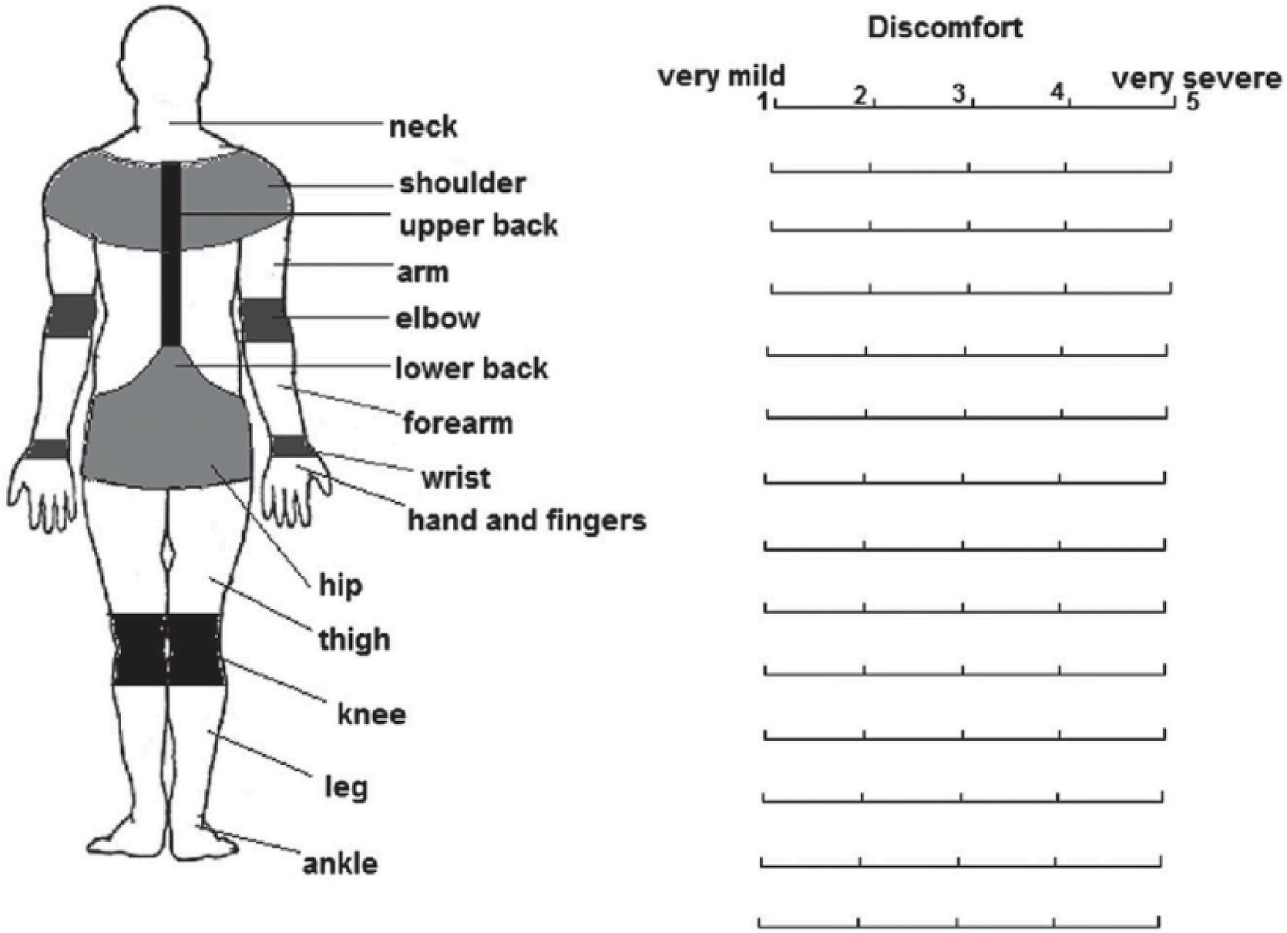


## Appendix Questionnaires

Data collection form

Prevalence of musculoskeletal disorders in dental practitioners of northern region in India.

DATE:

PLACE:

General information:

1. Age (Years),
2. Gender-
3. Weight(kg)
4. Height(cm)-
5. Dominant hand- Right/Left
6. Any history of (Musculoskeletal Disorders) MSDs? Yes/No

If Yes –specify the body part affected- … … … … … … ….

7. Any other disease?
8. Marital status-
9. Smoking?
10. Alcoholism?
11. Physical Recreational Activity, if Yes-Specify?... …. …. …. …. ….

Profession related information

1 Qualification- BDS/MDS/Dental Hygienist Course
2. Currently working as: UG Student/PG Student/Intern/Dental Hygienists/Professor/Dentist
3. Specialization (if MDS) - Prosthodontics, Periodontics, Endodontics, Orthodontics, Pedodontics, Oral Medicine & Radiology, Community Dentistry
4. Years of Practice till now (years; including BDS internship)-

### Work environment

Standard nordic questionnaire.

Likert scale rating form.
